# A chronology of global air quality

**DOI:** 10.1098/rsta.2019.0314

**Published:** 2020-09-28

**Authors:** David Fowler, Peter Brimblecombe, John Burrows, Mathew R. Heal, Peringe Grennfelt, David S. Stevenson, Alan Jowett, Eiko Nemitz, Mhairi Coyle, Xuejun Lui, Yunhua Chang, Gary W. Fuller, Mark A. Sutton, Zbigniew Klimont, Mike H. Unsworth, Massimo Vieno

**Affiliations:** 1 Centre for Ecology and Hydrology, Penicuik, UK; 2 School of Energy and Environment, City University of Hong Kong, Kowloon, Hong Kong; 3 Faculty of Physics and Electrical Engineering, University of Bremen, Bremen, Germany; 4 School of Chemistry, The University of Edinburgh, Edinburgh, UK; 5 IVL Swedish Environmental Research Institute, Stockholm, Sweden; 6 School of GeoSciences, University of Edinburgh, Edinburgh, UK; 7 The Boundary, Goodley Stock Road Crockham Hill, Kent, UK; 8 Environmental Science and Engineering, China Agricultural University, Beijing, People's Republic of China; 9 Nanjing University of Information Science and Technology, Nanjing, Jiangsu, People's Republic of China; 10 Imperial College London, London, UK; 11 International Institute for Applied Systems Analysis (IIASA), Laxenburg, Austria; 12 Oregon State University, Corvallis, OR, USA

**Keywords:** acid rain, air quality, ozone, eutrophication

## Abstract

Air pollution has been recognized as a threat to human health since the time of Hippocrates, *ca* 400 BC. Successive written accounts of air pollution occur in different countries through the following two millennia until measurements, from the eighteenth century onwards, show the growing scale of poor air quality in urban centres and close to industry, and the chemical characteristics of the gases and particulate matter. The industrial revolution accelerated both the magnitude of emissions of the primary pollutants and the geographical spread of contributing countries as highly polluted cities became the defining issue, culminating with the great smog of London in 1952. Europe and North America dominated emissions and suffered the majority of adverse effects until the latter decades of the twentieth century, by which time the transboundary issues of acid rain, forest decline and ground-level ozone became the main environmental and political air quality issues. As controls on emissions of sulfur and nitrogen oxides (SO_2_ and NO*_x_*) began to take effect in Europe and North America, emissions in East and South Asia grew strongly and dominated global emissions by the early years of the twenty-first century. The effects of air quality on human health had also returned to the top of the priorities by 2000 as new epidemiological evidence emerged. By this time, extensive networks of surface measurements and satellite remote sensing provided global measurements of both primary and secondary pollutants. Global emissions of SO_2_ and NO*_x_* peaked, respectively, in *ca* 1990 and 2018 and have since declined to 2020 as a result of widespread emission controls. By contrast, with a lack of actions to abate ammonia, global emissions have continued to grow.

This article is part of a discussion meeting issue ‘Air quality, past present and future’.

## 1. Introduction

The potential subject area is large and the focus here is on the chronology of air pollution by human activity, identifying the main issues, their causes and the regional and global trends. Other papers in this volume, to which links are made, provide the wider context, the policies developed to address the problems and the possible futures.

There are four rather different sources of evidence to provide the narrative for this account. These include written documents including early legislation, direct measurements of atmospheric composition, chemistry transport models, which simulate atmospheric composition changes from a knowledge of emissions, meteorology and chemical processing of pollutant gases, and, finally, remote sensing of the atmosphere from aircraft and space. The early documents are fascinating and provide hints at the underlying chemistry, but are entirely lacking in quantitative detail. Legal documents indicate the intent, but, for reasons elaborated later, did not significantly constrain the developing global issues until the later decades of the twentieth century. High-quality measurements of air pollutants are restricted to the last 150 years and numerical modelling to the last 40 years, leaving considerable scope for speculation on the early trends. There is necessarily some subjectivity in the selection of information sources used to describe the air pollution chronology outlined here, as summarized in table 1. For this purpose, we have focused on what we consider to represent major milestones based on: (i) the recognition of key aspects of air pollution, (ii) of quantitative evidence and (iii) of major points of changes in air pollution levels. Other perspectives on the topic have been provided by Colbeck [5] and Mosley [6]. The main air pollutants of interest examined here are sulfur dioxide (SO_2_), nitrogen oxides (NO*_x_*), ammonia (NH_3_), volatile organic compounds (VOCs), primary particulate matter (PM), and their reaction products, including fine particulate matter (PM_2.5_) and tropospheric ozone (O_3_).

**Table 1.** Components of the selected chronology of air pollution presented in this paper.

date	air pollution event
400 BCE	The relationship between air and health developed as an important part of the book *Airs, waters and places* attributed to Hippocrates
first century AD	Writers from imperial Rome, e.g. Seneca and Frontinus, refer to the probable health impacts of smoke
947–1279	Smoke and gaseous pollutants from coal burning identified as a problem in Central Asia by Al-Mas'ūdī (947) and in China during the Song Dynasty (960–1279)
1273	The Smoke Abatement Act, the earliest legislation in England, prohibits use of coal as it is ‘prejudicial to health’
1610	*The Law of Nuisance (UK)*: *William Aldred's pig farm* case
1661	John Evelyn published *Fumifugium or The Inconvenience of the Aer and Smoak of London*
seventeenth century	Harmful effects of air ascribed to various components, e.g. Kenelme Digby (acids), Nehemiah Grew (lead), John Evelyn (sulfur) and John Hall (antimony or mercury)
eighteenth century	Guillaume François Rouelle detects SO_2_ by absorbing the gas in strong alkalis; Carl Wilhelm Scheele detects NH_3_ via absorption with acids
1872	Robert Angus Smith publishes *Air and Rain: The Beginnings of a Chemical Climatology*, having undertaken the first multisite, multipollutant measurements
1878	The UK Royal Commission on Noxious Vapours
1894	The ‘great horse manure crises’ of London and New York
1905	Smoke Nuisance Acts in Bengal 1905
1952	The Great London Smog; 12 000 die in two weeks [1] Los Angeles smog, chemistry and effects described [2]
1956	The UK Clean Air Act
1960	Extensive local ecological damage by smelters (e.g. [3])
*From 1967, air pollution problems are recognized as international issues*	
1960s	Acid rain extensively described by Svante Oden
1972	United Nations Stockholm Conference confirms acid rain as an important international issue in Europe
1970s	Ground-level ozone threat to ecosystems identified in North America and Europe following earlier concerns of effects of the ozone on human health
1977	USA establishes its National Acid Deposition Program (NADP)
1979	UNECE Convention on Long Range Transport of Air Pollution (LRTAP) established
1980s	Forest decline recognized in Europe and North America
1985	Helsinki Protocol: Commitment to reduced SO_2_ emissions by 30% (The 30% club)
1980s–1990s	Eutrophication of ecosystems by nitrogen deposition recognized
1991	Canada-USA Air Quality Agreement
1993	The ‘Six Cities’ study in North America re-focuses attention on the human health effects of air pollution PM_10_
1995	Launch of the first satellite for passive remote sensing atmospheric composition (GOME) for global ozone monitoring [4]
1999	The UNECE Gothenburg Protocol adopted to tackle multipollutant multieffects (acidity, ozone and eutrophication)
2000s	Emissions of SO_2_ and NO_x_ in Asia increasingly dominate global emissions and adverse effects
2010	Widespread evidence of recovery from effects of acid deposition in Europe and North America with the decline in emissions of SO_2_ and NO*_x_*
2012	Beijing smog, 13th January, with concentrations of PM and SO_2_ similar to London 1952
2015	Global SO_2_ emissions reduced by 15% from the 1990 peak, while all other air pollutants still increasing
2018	Emissions of SO_2_ and NO_2_ declining rapidly in China
2018	Peak global NO*_x_* emission? Global emissions of NH_3_ and VOC continue to rise
2020	COVID-19: The global pandemic dramatically reduces emissions of industrial- and transport-related emissions of SO_2_, NO*_x_*, VOC and primary PM

Clear written evidence shows that early society recognized a threat to human health and the wider environment from air pollution. However, the identity of the gases and particles remained largely unknown, and there were no measurements to quantify the problem. Early attempts to regulate emissions show that the lawyers in their day clearly had the means to articulate societal desire for a cleaner environment, but the laws developed were not supported by the infrastructure necessary to make them effective. The lack of consistent language describing the underlying science also makes the early literature difficult to interpret from a twenty-first century perspective.

The early history takes us to the period of elucidation of the compounds present in the atmosphere and to early measurements, mainly in the seventeenth to nineteenth centuries, following which direct measurements began in earnest. Sporadic measurements of air quality began in the late nineteenth century, especially by Robert Angus Smith in the UK [7], the first scientist to attempt multisite, multipollutant investigations of the chemical climatology of the polluted atmosphere. The early developments in understanding of air pollution were mainly by chemists, who continued their leadership of the mechanistic underpinning of the science through the twentieth century (e.g. [8,9]). Distributed sites to measure atmospheric composition gradually developed through the mid-twentieth century and by the time acid rain became a focus of scientific and political interest in the late 1960s there were networks in Europe and North America to study the composition of air and precipitation at regional scales (e.g. [10,11]). In addition, local pollution problems in industrial cities, mainly in Europe and North America, and around notable point sources, provided early measurements of large local effects by some of the main pollutants.

The ground-based monitoring networks in place by the year 2000 (table 1) included regional and global air chemistry measurements. The third main source of time-series data to assess the chronology of air pollution is the application of chemistry transport models (CTMs) with global meteorological models and spatially disaggregated inventories of pollutant emissions. The final source of data is that provided by satellite remote sensing, which has developed over the last three decades, providing global concentration fields for the major air pollutant gases (SO_2_, NO_2_, NH_3,_ CO, and O_3_).

These complementary sources are used here to provide a summary of the development of specific air pollution issues through the late nineteenth and early twentieth centuries and in the last two decades revealing some important signs of recovery from effects of air pollution in Europe, North America and East Asia.

## 2. Pre-1750 early evidence air pollution posed a risk to human health and ecosystems

Early humans would have been aware of at least some of the potential hazards in the air they breathed from their general discomfort in the presence of smoke and combustion gases close to open fires. The need for shelter and warmth led to fires inside shelters, and in confined structures, the exposure to potentially toxic gases and particles is considerably enhanced. Given the directly noxious properties of many combustion products (smell, and lachrymose and respiratory effects), it is surprising that so many societies had dwellings with open fires and no chimneys. The development of the chimney itself can be seen as a key milestone for indoor air quality, adopted at first in the largest houses from the twelfth century [12]. Today, indoor air pollution is an important contributor to effects on human health. All subsequent analysis here, however, is devoted to the outdoor environment.

Evidence from Greece shows that the problems of polluted air outdoors were being documented at least 2400 years ago. The book *Airs, waters and places* attributed to Hippocrates (*ca* 400 BC) suggested all sorts of illness as being related to the quality of air. The worst it seems was in cities facing damp westerly winds, where the inhabitants ‘are likely to have deep, hoarse voices, because of the atmosphere, since it is usually impure and unhealthy in such places' ([13], p. 83). Writers a little later from Imperial Rome understood the probable health impacts of smoke with Seneca (*ca* AD 63–65) referring to the problem and Frontinus (*ca* AD 96) proudly declaring how his contribution to aqueducts and fountains has helped make the air purer: ‘the causes of the unwholesome atmosphere, which gave the air of the City so bad a name with the ancients, are now removed’ ([14], p. 417). As Seneca recorded of a health break from Rome:

As soon as I escaped from the oppressive atmosphere of the city, and from that awful odour of reeking kitchens which, when in use, pour forth a ruinous mess of steam and soot, I perceived at once that my health was mending… So I am my old self again, feeling now no wavering languor in my system, and no sluggishness in my brain ([15], p. 193).

It is notable that the reference to the brain matches an effect of ammonium-containing air pollution from naturally burning coal caves along the Silk Road in Central Asia as later recorded by the Arab geographer Al-Mas'ūdī [16]. A book by Shen Kuo (1031–1095) written during the Song Dynasty (AD 961–1279) provides further evidence of concern in China about air pollution from coal burning [17]. Other post-classical writers, especially in the Arab world, contributed observations about air pollution during the ‘Dark Ages’ when considerable learning was being lost in Europe [18]. Ultimately, however, little changed throughout the Middle Ages in the understanding of the causes of disease and possible role of air pollutants reflecting the persistence of the classical miasmatic concept that odours and other matter in air were the controlling influences for human health [19], an idea going back to the time of Hippocrates.

**Figure 1.**
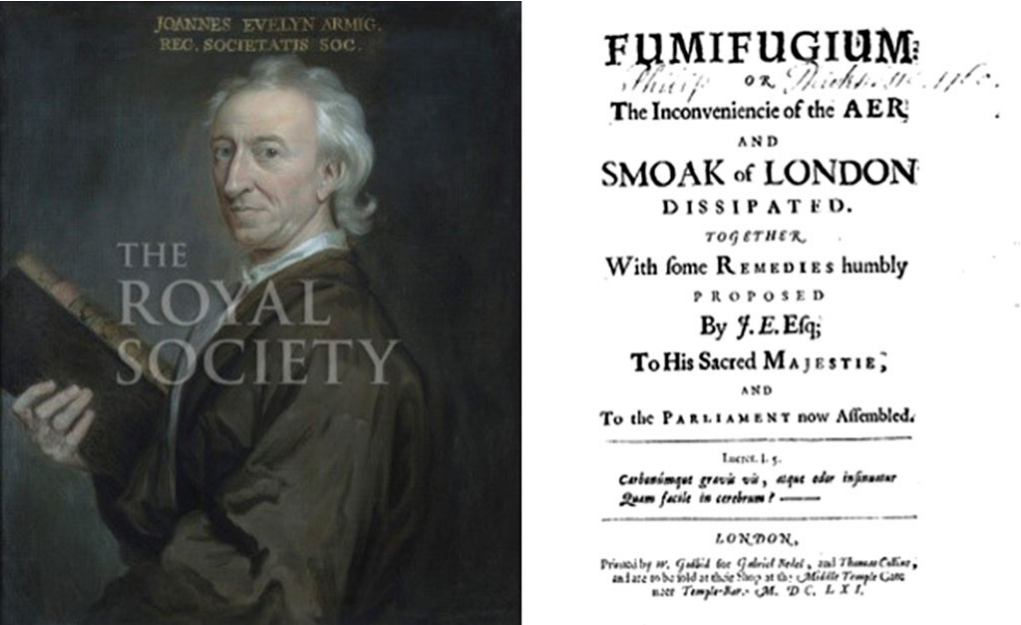
John Evelyn and the title page of *Fumifugium* (1661). (Online version in colour.)

In the seventeenth century, John Evelyn published *Fumifugium or The Inconvenience of the Aer and Smoak of London* [20] (figure 1). This iconic document described air pollution in London and suggested ways of reducing the scale of the problem. He proposed moving industries including brewing and lime-burning to the countryside, well outside the city. John Graunt, a contemporary of Evelyn, suggested a correlation between rates of mortality and pollution, especially in fog episodes [21]. In the absence of any chemical data, or indeed any numerical values to quantify the pollutants present, we have only the narrative, but it clearly identifies a serious problem for human health. Evelyn wrote of London in 1661: ‘that this glorious and ancient city should wrap her stately head in clouds of smoke and sulphur, so full of stink and darkness’.

There was some recognition of the strong-smelling sulfur pollutants derived from coal or industrial processes such as discussed in *Fumifugium* and also in Shakespeare's observation about the reek of lime-kilns (*The Merry Wives of Windsor*, Act III, Scene 3). Lime-kilns were used extensively in Europe since Roman times and were a noted source of air pollution. There was little understanding of atmospheric chemistry, however, although scientific interest became more important by the mid-1600s [19], with the harmful effects of air pollutants ascribed to various components of the air by Kenelme Digby (acids), Nehemiah Grew (lead), John Evelyn (sulfur) and John Hall (antimony or mercury).

## 3. The development of laws to control air pollution 1273–1900

The earliest legislation in England was the 1273 Smoke Abatement Act, prohibiting the use of coal as it was ‘prejudicial to health’ [22]. Some mediaeval societies approached air pollution control by keeping the sources outside the city walls, a concept found in Aristotle's *Athenian Politics* and in ancient Roman regulation [23]. This practice continued in mediaeval Europe, but also Asia, notably in relation to the extensive fifteenth-century Thuriang pottery kilns, which were located in the northern lee of Si Satchanali, Thailand. These early examples of what we may now like to call ‘environmental law’ include controls on the burning of sea coal and the ‘forestry laws’ (protecting the various species of game living in the forests). Most of these examples derive from the particular whims and prejudices of individual rulers, often heavily influenced by those within the upper social echelons of society. There was no modern science involved: the problem was perhaps a visual blot on the monarch's landscape, an appalling smell or a passion for hunting. In every case, the control or prohibition was imposed without any need to resort to the scientific knowledge base of the time.

In the Western world, one has to look to the Renaissance and the subsequent Reformation as forming the basis from which arose our modern methodologies for scientific enquiry. In the UK, this change in methods of enquiry alongside the rise of industrialization in the latter part of the eighteenth century and its increasing pace in the nineteenth century enabled the likes of David Hume (1711–1776), Jeremy Bentham (1748–1832) and John Stuart Mill (1806–1873) to articulate social philosophies such as utilitarianism—philosophies which ultimately gave momentum to centralized regulation of what we would now describe as ‘environmental issues'.

The direct intervention of the British Government by way of legislation was limited throughout this period. The Alkali Works Regulation Act 1863 and its Alkali Inspectorate were the prime example of governmental responsiveness to environmental matters during this period: necessity, driven by widespread and self-evident health and welfare problems, but enacted reluctantly.

Initially, it was the ‘common law’ that was used to combat instances of polluting activity in England. The English ‘civil claims procedure’ requires a complainant, a defendant and proof on a balance of probabilities that the ‘injury’ complained of was caused by the action or inaction of the defendant by reason of breach of a standard of care—a standard established and refined by the judiciary over many decades on a case-by-case basis. This was thereby inevitably a standard that was intrinsically susceptible to circumstance and prevailing norms. This reliance on an ‘after-the-fact’ procedure, coupled with a requirement to establish a clear, legally recognizable causal link between alleged cause and supposed effect, was to permeate the UK's approach to what we now term environmental regulation. The approach was one of future prevention of what could be shown to have already been clearly unacceptable rather than a ‘precautionary approach’, the latter now underpinning much of current thinking in relation to matters of the environment and social well-being generally.

A key step on the way to developing a UK legal framework for air pollution is the ‘The Law of Nuisance’. As early as 1610, *William Aldred's* case, as it is known, saw the courts intervene against one Thomas Benton for building a pigsty ‘so near the house of the plaintiff that the air therof was corrupted’. The court found that light and clean air were considered necessary for wholesome habitation. In discussing the issues raised, the court drew a distinction between ‘trifling inconveniences' that made life inconvenient or uncomfortable—when location could be a legitimate consideration in making such a finding—and material damage to property that diminished its value when location was largely irrelevant. The overall result was that the common law proved increasingly inadequate to address the sorts of issues that social philosophers and reformers were coming to focus on as increasing urbanization gave rise to self-evident public health and welfare issues.

Between 1800 and 1850, the population in England and Wales doubled to 16 million and doubled again by 1900, accompanied by dramatic changes in the distribution and concentration of the population as industrialization drew people from rural areas to what soon became highly urbanized areas with insanitary housing, disease, noxious emissions and fossil fuels adding to the toxic mix. The ‘Royal Commission on Noxious Vapours’ of 1878 recorded many examples of the kinds of damage resulting from what at that time was uncontrolled industry. However, the UK government was slow to take remedial action because of the importance of industry to the national economy. Despite the manufacturing controls introduced in the Alkali Act 1863, and the establishment of an Alkali Inspectorate, the increasing number of alkali works (manufacturing sodium carbonate, while emitting hydrochloric acid as air pollution) meant that the UK experienced no meaningful decrease in emissions of pollutants. Legislation requirements to use ‘best practicable means', together with a piecemeal approach, exacerbated what was in any event often indifferent enforcement of the legislation.

Nevertheless, laws to control air pollution that are recognizably modern did develop through the latter part of the nineteenth century, and these also reflected the sanitary reform that characterized the broad public health concerns of the time [24]. As might be expected, they were common in Europe and North America, but also followed imperial administrations across the world, so were well known in India (e.g. Smoke Nuisance Acts in Bengal 1905 and Bombay 1912) and Hong Kong. The wide range of international law was reviewed at the London Public Health Congress in 1905, often cited as the place where Henry Antoine Des Voeux coined the term ‘smog’ [25]

## 4. 1750–1950 Urban air quality and the industrial revolution

During the early phase of the industrial revolution, beginning in the UK in the late eighteenth century and spreading through Europe and North America, a rapid growth in coal combustion in the developing cities substantially increased emissions of SO_2_, NO_2_, NH_3_ and smoke (e.g. [26,27]). The problem of air pollution focused in this period on human health. In part, emissions were due to industrial development and rapidly increasing emissions from short stacks. Additional sources were from domestic emissions by the rapidly growing urban population of factory workers who mostly burned coal for warmth and cooking. Ambient concentrations were not measured during the eighteenth and early nineteenth centuries, and values are a matter of speculation.

Emissions from combustion were the main contributors to poor air quality, but they were not the only pollutants. It is important to mention emissions of NH_3_ from the large urban population of horses for transport, which would have added to NH_3_ released by coal combustion [16]. The quantity of horse dung on urban roads was recognized as a growing problem in the late nineteenth century, for example 100 000 horses in New York producing 1000 tonnes of manure daily (the ‘great manure crisis’ in New York and London; [28]), with a major problem projected into future decades. Prior to the twentieth century, horse populations were substantial in all major cities. The poor state of sewage treatment, especially during the rapid expansion of the eighteenth and nineteenth centuries, also contributed to emissions of NH_3_. The rapid replacement of horse drawn transport by motor vehicles in the early decades of the twentieth century avoided the problems forecast for cities like London and New York. Little attention has been drawn to the combination of SO_2_, NO*_x_* and NH_3_ in the urban chemical climate of the nineteenth century, perhaps due to the lack of measurements and the focus on pollutants from combustion sources. But the presence of large emissions of NH_3_ would have promoted the formation of particulate (NH_4_)_2_SO_4_ [29] and the rapid deposition of SO_2_ to terrestrial surfaces [30]. Among the few early urban measurements, Smith [31], recorded concentrations of NH*_x_* (NH*_x_* is the sum of gaseous NH_3_ and particulate NH_4_^+^) in London of 80 to greater than 1000 µg m^−3^, with the highest values recorded during fog. The deposition of NH_3_ would also have contributed to changes in species richness of plant communities in urban areas [32].

The degradation of air quality during the period 1750 into the twentieth century was primarily in urban areas or close to large industrial point sources. Most major European cities in the late nineteenth century had air quality problems. London and Edinburgh, respectively known colloquially as ‘the Smoke’ and ‘Auld Reekie’, were notable but far from unique. All major cities of the UK suffered. Popular works of English literature by Dickens and Conan Doyle contain many descriptions of dense swirling smog contributing to an air of danger and gloom in Victorian London. Likewise, the major cities throughout Europe, where coal provided the main fuel for industry and domestic heating, developed similar air quality problems.

The pollutants from coal combustion included SO_2_, NO_2_, smoke and, to a lesser extent, HCl from the chlorine in coal [33,34]. Urban concentrations of SO_2_ and smoke in the large cities in the middle decades of the twentieth century were commonly between 50 and 100 µg m^−3^, and many UK cities had annual values in this range [35]. Meteorological conditions in the winter months leading to low wind speed and a cold surface air greatly reduce dispersion of pollution, and in these conditions, concentrations of smoke and SO_2_ could exceed 1000 µg m^−3^, as in the infamous 1952 London smog episode [19].

## 5. 1952 The Great London Smog

Air pollution was, until the 1950s, largely accepted as a consequence of industrial activity, with a perceived willingness to tolerate the grime, degraded visibility, erosion and blackening of valued buildings and effects on human health, agriculture and natural ecosystems. It took a major event to change the public and political perception of the problem and the need for control measures.

The 1952 London smog resulted in the premature mortality of approximately 12 000 people [1]. The public and then more slowly the political reaction led to the introduction of the Clean Air Act in 1956, some 3 years after the event. It arose from a Bill to the UK Parliament initially proposed by a back-bench Member of Parliament (Sir Gerald Nabarro), and not an initiative of the Government Ministers at the time, an indication of the prevailing focus on housing, industrial growth and recovery from the effects of the Second World War. The lack of prioritization for matters of the environment was a feature of 1950s Britain, where food rationing was still in place in 1952. However, this Act of Parliament was a very important step, eventually leading to widespread reductions in emissions of smoke and SO_2_ in urban areas.

**Figure 2.**
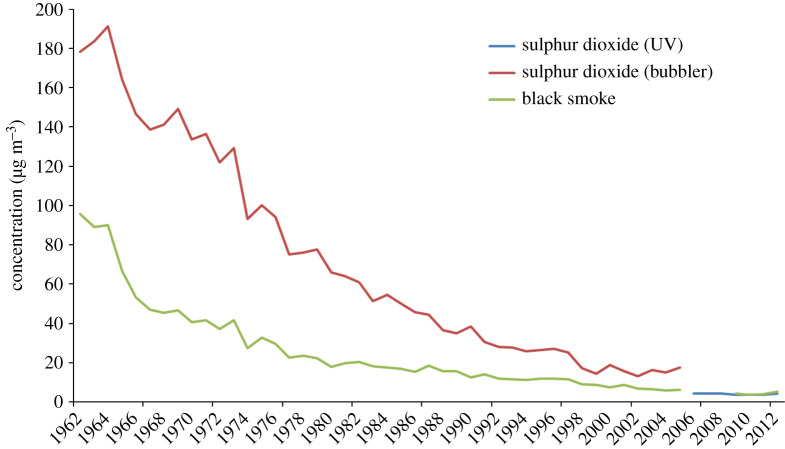
The decline in SO_2_ and smoke in London following the Clean Air Act (1956), including data from the ‘bubbler method’ sampling air through a peroxide solution in water and ultraviolet (UV) spectroscopy. (M. L. Williams, personal communication, 2017). (Online version in colour.)

During the three decades following the London smog, many urban power stations and other polluting industrial sources were closed, and new, larger, more efficient power stations were constructed in rural areas. These each typically produced 2000 MW output of electrical power and consumed 5 million tonnes of coal annually. UK emissions of SO_2_ continued to increase through the 1950s to a peak in the 1960s, mainly driven by industrial emissions and especially power generation. The new large power stations were equipped with reasonably effective controls for PM, but none of the new units had SO_2_ removing equipment until Drax in 1988 and Ratcliffe in 1995. The closure of the large number of smaller very polluting urban power stations and other industrial sources with short stacks further reduced emissions of SO_2_ and smoke in cities and contributed significantly to the improving urban air quality. Ambient concentrations of smoke and SO_2_ declined by 60% between 1962 and 1975 in London, nearly a quarter of a century after the event that tipped the scales in favour of effective action on urban air quality (figure 2).

**Figure 3.**
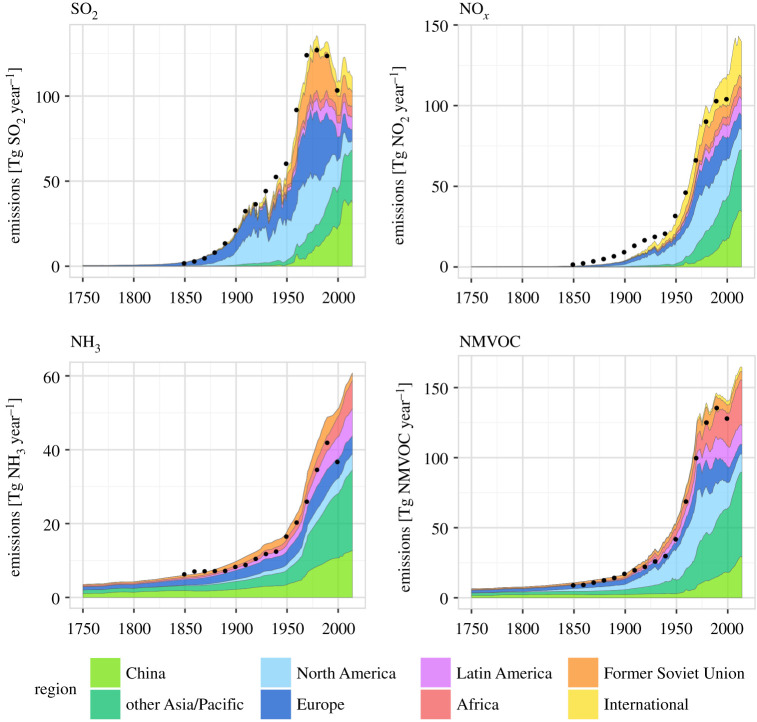
Global and regional emissions of SO_2_, NO*_x_*, NH_3_ and NMVOC between 1750 and 2010. Adapted from Hoesly *et al*. [37]. The dots show global estimates of an earlier study (CMIP5 [38]). (Online version in colour.)

Recognizing needs to reduce ground-level concentrations of SO_2_ and smoke, new power stations from the 1950s were built with increasingly high chimney stacks. The large rural power stations, many in the Trent and Ouse valleys of the English Midlands and industrial north, had larger stack heights, many at 200 m, promoting dispersion and reducing local effects [36]. Similar changes in power generation were taking place across Europe where SO_2_ emissions peaked in the 1980s [37]. It is notable that on a global scale, Europe and North America were responsible for most (greater than 80%) of the global SO_2_ emissions prior to 1970 (figure 3). Emissions in North America grew rather faster than those in Europe and with tall stacks also used in North America to disperse pollutants, to minimize local effects. Global emissions of NO*_x_*, non-methane volatile organic compound (NMVOC) and NH_3_ also increased rapidly during the late twentieth century (figure 3), as consequences of increased energy consumption, transport, solvents and agricultural activity. These graphs show the major shifts since 1980, where China and the Asia/Pacific region have replaced Europe and North America as the main global sources of air pollution.

## 6. The recognition of regional air quality issues and long-range transport of air pollution

Although there had been earlier concerns regarding ecological effects, the policies to control air pollution during the 1950s and 1960s were aimed at protecting human health, with a focus on urban air quality. But, as noted above, the gradual improvement of urban air quality took place while country-scale emissions of SO_2_ were close to their maximum, as a consequence of increasing emissions from large combustion plants with tall chimney stacks. During this period, annual UK emissions of SO_2_ reached their peak of 3 Mt S annually [39]. Annual European and North American emissions of SO_2_ also peaked during this period at 32 Mt-S [40] and 31 Mt-S [41], respectively.

The scale and effects on the countryside from the high levels of SO_2_ (and NO_2_) were not immediately recognized as an issue. In the Pennine hills between Sheffield and Manchester, the Forestry Commission was unable to establish plantations of Scots Pine due to the ambient exposure to SO_2_ and large areas of central England as well as all of the major cities were devoid of lichen species sensitive to SO_2_ [42]. Effects on agricultural crops were substantial, with some cultivars of grass developing resistance to SO_2_ [43], but knowledge of these domestic problems of air pollution was insufficient for the UK Government to introduce further legislation to reduce emissions. The Clean Air Act was regarded as adequate, and there were no limits on the overall scale of SO_2_ emissions, just a requirement to use stack heights tall enough to minimize the concentrations downwind at the surface following the philosophy of Best Practical Means [44]. Policy priorities at this stage were firmly focused on human health.

**Figure 4.**
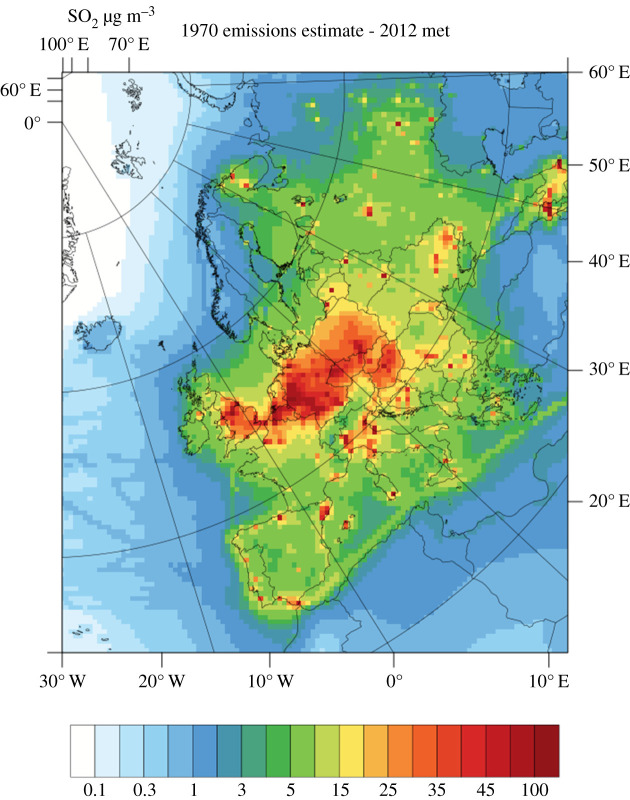
Annual mean European SO_2_ concentrations (µg m^−3^) in 1970, at around the time of peak SO_2_ emissions, modelled using EMEP4UK with 1970 emissions and 2012 meteorology (M. Vieno *et al*., personal communication, 2020). (Online version in colour.)

Elsewhere in continental Europe, tall stacks and large power plants located outside the cities were also regarded as effective policies to minimize effects on human health. Areas highly polluted by SO_2_ were extensive over England, Germany, eastern central Europe and the Low Countries (figure 4). Ecological effects were not considered sufficiently important to introduce further control measures, even though it was known that some industrial processes, especially smelting, produced striking examples of local damage from SO_2_ and metal deposition. For example, emissions from the Sudbury smelter in eastern Canada during the early twentieth century caused extensive areas of natural vegetation to be destroyed by the combination of exposure to very large concentrations of SO_2_ and large deposition rates of a range of metals [45,46]. Smelters were present in many countries globally, and large exposures to SO_2_ and metal deposition were common in their proximity with examples in Slovenia, Peru, Canada, USA, Russia, China, France, Poland and Zambia [47].

## 7. Early evidence of air pollution transport from measurements

There were seventeenth-century analyses of rainfall, possibly the first by Ole Borch in Denmark. These became more commonly undertaken by agriculturalists during the 1800s [19] and increasingly used worldwide [10,48,49], providing early evidence of inter-country exchange of pollutants from observations of contaminated snowfall. The deposition of urban sulfate in London rainfall was determined by Robert Angus Smith in 1869–1870 [7]. The number of estimates of the concentrations of substances in air increased rapidly through the nineteenth century. Russell [50] measured total PM gravimetrically in central London at 120, 360 and 860 µg m^−3^ in fine, dull and foggy weather, respectively [51]. Although measurements of carbon dioxide were frequent (as listed in Callendar [52]), this occurred only occasionally for trace gases such as ammonia [31,53]. The early twentieth century saw the development of the deposit gauge that measured wet deposition and whatever else fell into the large glass bowl [54] and the use of lead peroxide candles to determine deposited sulfur dioxide [55]. The widespread occurrence of SO_2_ led to increasingly sophisticated methods that involved drawing air through Dreschel bottles (bubblers) containing solutions of iodine, hydrogen peroxide or disulfitomercurate [56].

Table 2 shows the dates when regional networks to measure atmospheric composition were introduced, revealing that only the European Air Chemistry Network (EACN) was in place prior to the peak in European emissions of SO_2_. At the global scale, monitoring of global trends in background atmospheric composition is now coordinated through the Global Atmospheric Watch (GAW) network [58], whose origins can be traced back to the Background Air Pollution Monitoring Network (BAPMoN) first established in 1974 [59].

**Table 2.** Long-term monitoring activities in relation to acid rain and other pollutants (adapted from Grennfelt *et al*. [57]).

activity and time	geographical coverage and number of sites	programme centre	web page comments
atmosphere			
EACN (IMI network) 1955–1976	Europe >100 sites	Stockholm University	some sites continued within EMEP after 1976 (L. Granat, personal communication, 2019)
WMO GAW/BAPMoN 1964–	global >200 sites	World Meteorological Organization	http://www.wmo.int/pages/prog/arep/gaw/gaw_home_en.html
EMEP 1977–	Europe and ECE region of Asia approximately 350	Norwegian Institute for Air Research (NILU)	http://www.emep.int/
NADP 1977–	USA approximately 260 sites	University of Wisconsin-Madison	http://nadp.slh.wisc.edu/
CAPMoN (incl. APN) 1978–	Canada approximately 25 sites	Environment Canada	https://www.canada.ca/en/environment-climate-change/services/air-pollution/monitoring-networks-data/canadian-air-precipitation.html
EANET	East Asia	Asia Center for Air Pollution Research (ACAP)	http://www.eanet.asia/
Male Declaration 2003–	South Asia 15 sites	Originally the South Asia Cooperative Environment Programme; now Asian Institute of Technology	http://www.sacep.org/programmes/male-declaration http://www.rrcap.ait.asia/male
Ecosystems			
ICP Forests 1985–	Europe 5000 plots and 500 intense plots	Thünen Institute of Forest Ecosystems	http://icp-forests.net/
ICP Waters 1985–	Europe and North America approximately 250 sites	Norwegian Institute for Water Research	http://www.icp-waters.no/
ICP Material	Europe and North America approximately 40 sites	Rise KIMAB AB, Sweden	http://www.corr-institute.se/icp-materials/web/page.aspx
ICP Integrated Monitoring	Europe approximately 50 sites	Finnish Environment Institute	http://www.syke.fi/nature/icpim
ICP Vegetation	Europe	Centre for Ecology & Hydrology, UK	https://icpvegetation.ceh.ac.uk

## 8. Long-range transport of air pollution

The Norwegian playwright Henrik Ibsen's play ‘Fire’ (Brand) showed that people were aware of long-range transport of pollutants in the nineteenth century [60]:

Worse times, worse sins through the night of future flashes of Britain's suffocating coal dust is slowly descending over the countryside soiling all that is green strangling all that strives to grow creeping low and mixed with poison stealing sun and light from the valley pelting down as rain of ashes.

Even in the early 1950s, it was known that the majority of UK sulfur emissions were exported from the UK coastline, as Meetham [61] demonstrated using national monitoring data and a simple atmospheric mass balance. However, the magnitude of the effects of long-range transport of air pollutants from the major emitting countries in Europe on the net importing countries was not considered an important issue until the late 1960s and 1970s. The potential for transboundary transport within Europe can be readily visualized from the data in figure 4, given typical boundary layer wind-speeds of 10 m s^−1^ and an atmospheric e-folding lifetime for SO*_x_* of a few days.

## 9. 1960s Acid rain

Swedish scientist Svante Oden initially advanced the idea that the long-range transport of sulfur and acidity in Europe from the major sulfur emitting countries (UK, Germany, France and Poland in particular) was responsible for widespread acidification of freshwaters and loss of fish populations in Scandinavia [62]. Monitoring data for air and precipitation established by Egner and colleagues from 1955 (the EACN) provided the vital chemical data showing both the geographical patterns and trends in concentrations of the major ions in precipitation (e.g. [10]). The EACN data showed acidity and sulfate in precipitation increasing steadily through the 1950s and 1960s [63]. Oden attracted considerable Swedish interest with these ideas, much of it critical. The ideas were new and many aspects of the atmospheric chemistry and physics of the compounds involved were poorly understood. However, the evidence was persuasive, and further analysis by Swedish colleagues provided strong support for his arguments. A United Nations conference on the Human Environment in Stockholm in 1972 advanced the wider case that the pollution of one country by another through emission, atmospheric transport and deposition was unacceptable [64], building on the evidence of long-range transport of sulfur compounds within Europe and the effects in Scandinavia.

The Stockholm Conference of 1972 was a turning point in environmental science. There is little doubt that long-range transport and effects of pollutants had been taking place for decades; in fact, many had noted the inter-country transport of pollutants in Europe (e.g. [60]). It was only following Oden's analysis that international scientific and political attention was drawn to the subject and monitoring and process studies demonstrated the scale and ecological significance.

The initial reaction of the major polluting countries in Europe was mixed. All recognized the need to quantify the scale of inter-country transport of the major pollutants, the underpinning atmospheric chemistry and physics, and the effects on ecosystems. However, the legal instruments needed to be developed, and the supporting monitoring and analysis tools were still lacking. The industrial nations had designed tall stacks to disperse the pollutants without considering possible effects outside their jurisdiction. The combination of the science and political background is described in detail by Grennfelt *et al*. [57]. An earlier description of the political background in the UK by Rose [65] provides further insights and is typical of the large number of publications on the politics of acid rain. Research and monitoring activities expanded across Europe and the essential details of the emissions, atmospheric chemistry and deposition were presented at Dubrovnik in 1977 [66].

The first major international conference on acid rain was held in Columbus, OH, USA in 1975, beginning an important series of meetings on the subject approximately every 5 years from 1975 to 2015. Table 3 provides links to this series of meetings which shows the developing global scale of air pollution issues through the latter decades of the twentieth century, beginning with acid rain. The results of the Norwegian SNSF research programme into the causes and effects of acid rain were presented at the second international Acid Rain conference in Sandefjord in 1980 [68]. Clear links between sulfur emissions in the industrial nations of Europe and long-range transport to, and deposition and effects within, Norway and more widely in Scandinavia were demonstrated. The effects were primarily on freshwaters, with large declines in fish populations in the most acidified regions. The early studies in Scandinavia did not show negative effects on forests of the long-range transport of pollutants. Discussion meetings at the Royal Society of London reported the Pathways of pollutants in the atmosphere in 1979 (*Phil. Trans. A*, vol. 290) and Terrestrial Effects of deposited sulfur and nitrogen compounds in 1984 (*Phil. Trans. B*, vol. 305).

**Table 3.** The series of international conferences on acid deposition showing the broadening of issues and scale from 1976 to 2016.

date	issue	location	reference to proceedings
1976	acid rain	Columbus, OH, USA	Dochinger & Seliga [67]
1980	acid rain	Sandefjord, Norway	Drabløs & Tollan [68]
1985	acid deposition, forest decline	Muskoka, Ontario, Canada	Martin [69]
1990	acid deposition, eutrophication, ozone	Glasgow, UK	Last [70]
1995	acid deposition, eutrophication, ozone, critical levels	Gothenburg, Sweden	Grennfelt [71]
2000	acid deposition, eutrophication, ozone, recovery	Tsukuba, Japan	Satake [72]
2005	acid deposition, eutrophication, ozone, recovery	Prague, Czech Republic	Brimblecombe *et al*. [73]
2011	acid deposition, eutrophication, ozone, recovery	Beijing, China	
2016	acid deposition, eutrophication, ozone, recovery	Rochester, NY, USA	Aherne *et al*. [74]

By the late 1970s, the requirement to reduce European emissions especially of SO_2_, and thereby reduce deposition of acidifying compounds, in Scandinavia, was clear. This led to the establishment of the Convention on Long-range Transboundary Air Pollution (LRTAP) as a major international framework to address the problem [75]. This required extensive monitoring of the chemical composition of air and precipitation and associated meteorological variables, the development of atmospheric transport and deposition models to quantify the net transfer of pollutants between countries (the European Monitoring and assessment Programme (EMEP)), and a framework for interpretation and negotiation of the issues between the countries. The development of the LRTAP Convention has proved a very effective process to bring together the process-based science, the monitoring and the modelling (within the EMEP) and policy development, ultimately leading to international agreements to reduce emissions of SO_2_ and subsequently NO_2_, VOC and other air pollutants.

Like the human–health-oriented measurement programmes instigated in the USA, the LRTAP programme recognized the need to simultaneously measure a wide range of constituents, but also recognized the need to make measurements over long time periods to overcome the considerable inter-annual variability in meteorological conditions [76]. The LRTAP programme also introduced the explicit goal of making measurements to support associated atmospheric modelling.

In the early 1980s, a group of European countries considered a reduction in SO_2_ emissions appropriate, but in the absence of country-specific contributions to the ecological damage in Scandinavia, an arbitrary agreement to reduce emissions by 30% was proposed. Many, but not all countries, supported the measure, forming the 30% club. Arguments were presented that the costs of control were substantially greater than the benefits. For example, it was stated (incorrectly) that ‘acid deposition is a million dollar problem with a billion dollar solution’ [77]. Nevertheless, the 30% club was the basis for the first Sulphur Protocol, signed in Helsinki in 1985, which stipulated a reduction in sulfur emissions of 30% between 1980 and 1993.

Freshwater acidification and a decline in fish populations were the initial focus and the evidence that the cause was long-range transport of pollutants, mainly sulfur, was compelling. There was a secondary focus on the health of forests in Scandinavia but here the evidence was not persuasive.

The scientific and political interest in acid rain in Scandinavia and more widely in Europe stimulated interest elsewhere, especially North America, where similarly large increases in emissions of SO_2_ had occurred (figure 3). The USA had established its own National Atmospheric Deposition Program (NADP) in 1977. The combination of a large source area in the Ohio River valley and a large area downwind with geology and ecosystems sensitive to acidification soon led to the recognition of problems from long-range transport of pollutants similar to those identified in Europe. The fact that substantial areas of acid-sensitive ecosystems were located in Canada added a political dimension similar to that in Europe, with one country being responsible for ecological problems in a neighbouring territory.

## 10. 1980s Forest decline

By 1980 acid rain, or more correctly acid deposition, recognizing the importance of both wet and dry deposition to the total input to the ground [78], was established as an international issue, and all industrial countries engaged in research and many in the development of control measures.

Interest in acid deposition in Europe was greatly stimulated in the early 1980s by a decline in the health of forests in the most polluted regions [79]. The most damaged forests were those in the uplands in border regions of the Czech Republic, Poland and the German Democratic Republic where die-back of the forest was extensive. In parts of Germany, especially the Harz Mountains, the tree-line moved down the hills as damage at high elevation which was a feature of the problem progressed to lower levels. The large areas of forest decline throughout Germany (Waldsterben) became a defining environmental issue of the late twentieth century [32]. The causes of forest decline were hotly debated and contentious [79]. The main causal agents appeared to be acid deposition and ozone, but excessive nitrogen deposition and metals were also possible contributors and, at many of the sites of forest decline, exposures to large inputs of a combination of these pollutants were common. While many publications address the problem, there is no consensus to date on the proportions of the observed damage attributable to each of the pollutants and mechanisms of damage.

Forest decline was an important part of the acid deposition story in North America, and a particular species, red spruce, showed winter injury that was shown to be associated with the exposure to acidic cloud-water and sulfate in the Appalachians [80].

From the mid-1980s, sulfur and acid deposition declined steadily in Europe and North America, with recovery in atmospheric composition preceding any signs of ecosystem recovery [81,82]. While there were clear links between acid deposition and forest decline in both Europe and North America, ground-level ozone was also implicated in both regions [83,84].

## 11. Ground-level ozone

The broadening of the ecological focus from freshwaters to forests and the expansion of the number of pollutants implicated in effects was an important development. In expanding the range of effects and pollutants, the regional scale was also expanding considerably.

Ozone is formed within the atmosphere following photolysis of oxygen in the stratosphere, and some is transferred into the troposphere and contributes to ozone at the ground level [85]. However, ozone is also produced through the photochemical degradation of carbon monoxide and VOCs in the presence of NO_2_, and the issue of ozone in surface air is commonly referred to as ground-level ozone to distinguish it from stratospheric ozone issues. It was first recognized as a problem for human health and vegetation in California, and especially the Los Angeles basin, where it was first described by Middleton *et al*. [86]. The presence of ozone concentrations that posed a risk to vegetation and human health over Europe was demonstrated in the early 1970s [87]. Over Europe, the background concentration of ozone has increased by approximately a factor of two since pre-industrial times [88], and episodes of elevated ozone were shown to be widespread in Europe in the 1980s [89]. The effects of ozone on natural vegetation and crops are discussed by Stevens *et al*. [32] and Emberson [90], respectively. The discovery of damaging ozone concentrations in Europe and North America greatly increased the recognition of photochemical oxidants in regional air quality issues in the 1970s and 1980s. The focus of control measures therefore broadened from sulfur and nitrogen oxides to include VOCs in many other industrial countries [57].

The relatively long lifetime of ozone in the troposphere (approx. 20 days) and photochemical production over regional scales makes ground-level ozone a continental and hemispheric scale pollutant [91]. The industrial regions of Europe and North America experienced frequent summer episodes of ozone in the 1970s and 1980s with concentrations exceeding 200 µg m^−3^. The control measures to date have all been country or regional in scale, and while important progress has been made in reducing peak values, especially in California, but also across much of the USA and Europe, ozone remains a substantial threat to crops, natural vegetation and human health [32,90]. Methane is playing a major role in the formation of background ozone, and there is increasing interest in taking policy actions to control methane emissions as it is both a greenhouse gas and an ozone precursor [92].

## 12. 1990 Eutrophication: the effects of nitrogen deposition on ecosystems

As an understanding of acid deposition developed, and ground-level ozone was recognized as an additional regional-scale air pollution issue, the importance of nitrogen compounds grew. Nitrogen compounds were always a part of acid deposition, even when deposited in reduced form as NH_3_ or NH_4_^+^ in precipitation, as the protons generated in soil following microbial oxidation to nitrate create acidity [93]. However, the Netherlands and the UK were first to observe widespread changes in botanical species composition of heathlands [94]. It was soon shown that the changes were being driven by nitrogen deposition from the atmosphere and by ammonia in particular. As always in ecology, the story is a little more complex, as the replacement of heather-dominated heathlands by grassland in the Netherlands was mediated by the heather beetle, but the underlying driver of change was the deposition of nitrogen compounds from the atmosphere [95]. The eutrophication of ecosystems by nitrogen deposition has been shown to reduce species richness of grasslands over regional scales in Europe [96,97]. Close to livestock sources of ammonia, the changes in flora can be substantial [98] and the form of the nitrogen deposited has been shown to be an important factor in the scale of effects, with gaseous ammonia being more damaging to heather than wet-deposited NO_3_^−^ or NH_4_^+^ [16,99]. Similar effects of deposited nitrogen on ecosystems have been reported in North America and China.

The scale of effects of pollutants on ecosystems quantified at the turn of the twenty-first century showed that 24% of global forests were exposed to phytotoxic exposures of ozone [100]. The development of the Critical Loads approach and integrated assessment methods proved valuable instruments in the development of policies to maximize the ecological benefits of control measures within the LRTAP Convention [57].

## 13. 1990s Human health regains the focus of political attention on air quality

As noted above, the early evidence of air pollution effects were largely human health-related until the discovery of acid rain effects in Scandinavia in the late 1960s. The recognition of effects of long-range transport and the deposition of pollutants changed the scientific and, for a while, the political attention. The broadening of the science interest into ground-level ozone and eutrophication were important in the science and effects, and led to controls on the precursor pollutant emissions in Europe through LRTAP protocols. In North America, efforts to control the precursor gases followed a different control process, but achieved similar reductions in emissions over the longer term. Emissions of SO_2_ in Europe and North America have been reduced in 2016 by approximately 90% from their peak values in the 1970s and 1980s, respectively (figure 3).

However, in the early 1990s, a publication showing associations across six US cities between human mortality and morbidity and levels of air pollutants, especially PM, changed the political and scientific focus of effects [101]. Subsequent publications on human health effects of pollutants following similar epidemiological approaches revealed the scale of effects on human health throughout the developed and developing nations. Current estimates are that outdoor concentrations of PM_2.5_ alone are responsible for annual burdens of 4.2 million premature deaths and 100 million disability-adjusted life-years lost globally [102]. These publications showed air pollution to be one of the major global causes of premature mortality and drew attention to the human health effects of pollutants at much smaller concentrations than had been implicated in the London smog of 1952. This refocused scientific and political attention on air pollutants back to human health.

The underlying logic of the change in focus is understandable, given the large numbers of individuals and the societal costs of poor health and mortality. By comparison, effects of pollutants on natural ecosystems, which are always difficult to value, and on agricultural and forest crops are smaller in value than those on human health. For these reasons, ecosystem effects have become a secondary consideration for the policy makers. Human health has been the primary focus for the control of air pollution since the late 1990s. Clean air legislation in Europe, North America, Japan and other developed countries targets both ambient levels and emission sources. Nevertheless, the multi-impact effects of PM, NO_2_ and O_3_ on human health and managed and natural ecosystems mean that UNECE-LRTAP protocols still fulfil a crucial role [57].

## 14. Particulate matter

The chronology presented here describes the development of air quality issues as they arose rather than providing a narrative for each pollutant. However, it is important to draw attention to PM and its role in current air quality problems.

PM features in the earliest reports of air pollution, although terminology has been inconsistent and often poorly defined with terms including smoke, soot, fume, haze and dust, frequently used somewhat indiscriminately through the literature. PM, described in detail by Harrison [103], in this issue, refers to the sum of all solid and liquid particles suspended in air and is a complex mixture of size, spanning at least four orders of magnitude (1–10 000 nm) and with a large range of chemical composition. The latter reflects the wide variety of contemporary sources and very broadly comprises carbonaceous particles emitted directly from combustion, dusts from industrial processes and within-atmosphere conversions of inorganic (SO_2_, NO*_x_* and NH_3_) and organic (VOC) gases into PM.

PM is the main contributor to human health effects by some margin, and it is also the form in which most of the long-range transport of sulfur and nitrogen-containing pollutants occurs. PM contributes to changes in the Earth's energy balance both by absorption (e.g. black carbon) and by dispersion and reflection of radiation. Many of the links between air quality and climate change are therefore due to interactions between PM and the radiative balance and thus climate [104]. Similarly, many of the effects of pollutants on ecosystems are due to the deposition of PM either directly by dry deposition on foliar surfaces or through occult or wet deposition [105].

Smog includes both particulate and gaseous components, but the visibility effects are dominated by PM.

Given the contribution of PM to the chemical climatology of the atmosphere over the developed and rapidly developing countries and the contribution of PM to effects on human health, it is likely that PM will continue to dominate control measures for some decades to come.

## 15. 2010–2020 Air quality globally

Emissions of most primary pollutants have declined in Europe, North America and Japan from the 1990s until the present with the greatest progress in SO_2_, but even NO_2_ and VOC emissions have decreased more than 50% from their peaks in these regions. By contrast, during the period 1990–2010, emissions have increased in East and South Asia, and elsewhere, so that reductions in global total emissions, even for SO_2_, are modest, with a reduction of 15% from the peak in 1990 (figure 3) [37].

For NO*_x_* emissions, the global total continued to rise and all the reductions in emissions in Europe, North America and elsewhere have been counterbalanced by increases elsewhere and mainly in Asia (figure 3). For NH_3_ and VOC, the case is similar to that for NO*_x_*, with the global total steadily increasing [37].

The large increases in emissions of all primary pollutants in South and East Asia have been widely reported and described by Zheng *et al.* [106]. Air quality in Asian megacities shows values for PM, SO_2_ and NO_2_ in episode conditions that are similar to the highly polluted atmosphere of London in the smog episodes of the 1950s, for example the Beijing ‘haze’ events in January 2012.

**Figure 5.**
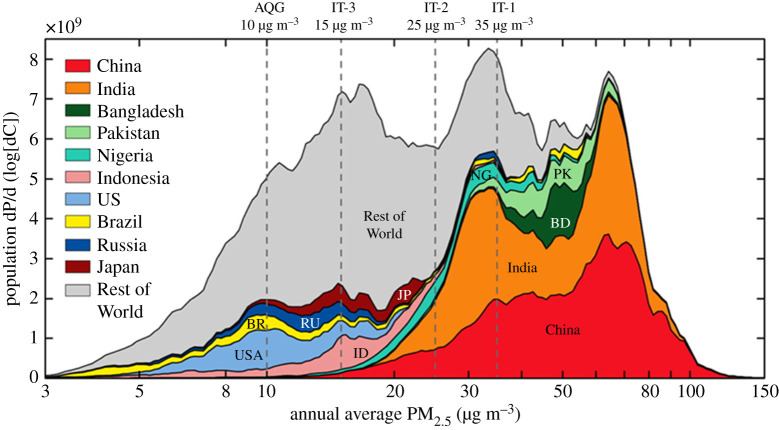
Distributions of the population as a function of annual (2013) average ambient PM_2.5_ concentration for the world's 10 most populous countries and the rest of the world. Dashed vertical lines indicate World Health Organization Interim Targets (IT) and the Air Quality Guideline (AQG). Source: Brauer *et al.* [107]. (Online version in colour.)

The global burden of air pollutants has therefore continued to increase into the first two decades of the twenty-first century. The focus of political attention remains firmly on human health due to the PM and NO_2_ exposure in urban areas of the developed and developing world. The distribution of ambient PM_2.5_ concentrations experienced by different regional populations presented in figure 5 shows how the current global air pollution health burden is disproportionately borne by countries in East and South Asia, rather than the countries that were afflicted in the early stages of the Industrial Revolution. Even so, the majority of the world's population live in locations where levels of ambient PM_2.5_ exceed the WHO guideline value.

It is important to note that this focus on human health deflects attention from the continued widespread exceedances of thresholds for effects of pollutants on managed and natural ecosystems [90].

## 16. Satellite remote sensing

The need to observe tropospheric pollution globally drove the development of passive and some active remote sensing techniques to measure the tropospheric burden of key pollutants trace gases and aerosol. In 1981, 1984 and twice in 1994 the MAPS (Mapping Pollution with Satellites), a gas correlation radiometer instrument flew for typically 9 day missions on the space shuttle and measured middle and upper tropospheric CO between approximately 55°N and approximately 55°S. A more advanced nadir gas correlation instrument MOPITT (Measurements of Pollution in The Troposphere) measuring in both the thermal and short-wave infrared has now made over 20 years of measurements of tropospheric CO and some CH_4_ from the NASA Terra, which was launched at the end of 1999.

The total columns and vertical profiles of ozone, O_3_, data products from NASA TOMS and SBUV on Nimbus 7 and later TOMS and SAGE II data [108,109] were used to retrieve O_3_, O_3_ amounts and distributions with a focus in the tropics.

From 1984, the SCIAMACHY (SCanning Imaging Absorption spectroMeter for Atmospheric CHartographY) project was developed and proposed to ESA in 1988. This led to the smaller GOME (Global Ozone Monitoring Experiment), being flown on ESA ERS-2 (1995–2011, [4]) and SCIAMACHY on ESA ENVISAT (2002–2012, [110,111]). Both satellites flew in polar orbits with equator crossing times of 10.30 and 10.00, respectively, and measured in nadir viewing geometry the upwelling radiation in the solar spectral region at the top of the atmosphere. The UV and visible nadir measurements of GOME and SCIAMACHY have been exploited to retrieve tropospheric columns of NO_2_, O_3_, SO_2_, HCHO, CHO.CHO, BrO, IO and H_2_O [4] in cloud-free regions and above clouds. The SCIAMACHY short-wave infrared spectral measurements enabled CO columns and for the first time the total dry column mixing ratios of CH_4_ and CO_2_ to be determined globally.

Satellite measurements revealed the growth in emissions in Asia and the declines in Europe and North America during the period 1996–2004 [112]. Satellite remote sensing in the solar spectral region also provides global fields for tropospheric SO_2_, CO, HCHO and CHO.CHO.

**Figure 6.**
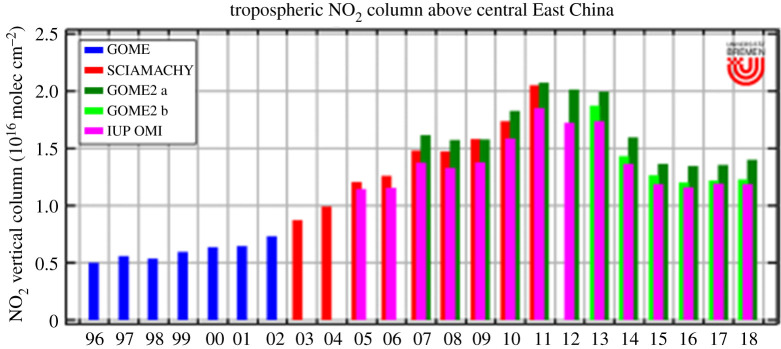
Trends in the tropospheric NO_2_ column over East China between 1995 and 2018 (A. Richter and J. P. Burrows, personal communication, 2020). (Online version in colour.)

The launch of the instruments AIRS on NASA and IASI, a CNES FTIR on EUMETSAT MetOp A B, has led to the detection of NH_3_ (see [113,114]). A combination of data from the GOME, SCIAMACHY and OMI instruments provides clear evidence of the increase in NO_2_ in Eastern China between 1995 and 2010 and the subsequent decline from 2010 to 2018, as shown in figure 6.

Satellite remote sensing also provides measures of PM, aerosol optical thickness, e.g. MODIS on NASA Terra (1999–present) and Aqua (2002–present) [107].

## 17. 2020 Are we at the global peak of air quality problems?

**Figure 7.**
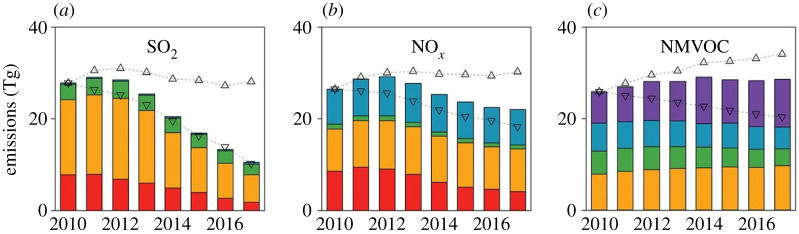
Annual emissions of (*a*) SO_2_, (*b*) NO*_x_* and (*c*) NMVOC in China between 2010 and 2017 (adapted from Zheng *et al*. [115]). (Online version in colour.)

Global emissions of sulfur have declined since the peak in 2000, and recent trends in China since 2012 show a reduction in emissions approaching 50% (figures 3 and 7). Such a reduction represents remarkable progress, relative to the time it took to reduce emissions in Europe and North America by a similar amount (approx. 20 years). Emissions of NO*_x_* in China have also declined, by approximately 25% over the last 8 years [115] and figure 6, although surface ozone has continued to increase [116]. It is therefore possible that the world has passed the point of maximum emissions of several major gaseous air pollutants as a combination of further controls in North America, Europe and East Asia drive down global totals. Climate change policies directed towards reduced use of coal and oil are expected to contribute further reductions in emissions of SO_2_ and NO_2_ over coming decades [117]. However, there are good reasons to be cautious, because emissions of ammonia, an important contributor to PM and eutrophication, continue to rise, and possible feedbacks between emissions of these gases and climate may drive overall emissions upwards [118,119]. Global emissions of CH_4_ and VOC also continue to rise, and in the case of biogenic emissions, it is possible that changes in climate and the widespread planting of new forests may accelerate global emissions of biogenic VOC (BVOC). Decisions over the species chosen for tree planting to increase carbon sequestration will also need to be made to simultaneously ensure that BVOC emissions do not increase. At present, it remains inconsistent in international policy that land use, land-use change and forestry are recognized as areas to count as carbon credits in the UN Framework Convention on Climate Change, but when it comes to the revised Gothenburg Protocol under the LRTAP Convention, the accompanying BVOC emissions are considered ‘natural’ and are excluded from the emissions commitments. Both the benefits for carbon and the possible disbenefits for BVOC will need to be recognized in future international agreements.

**Figure 8.**
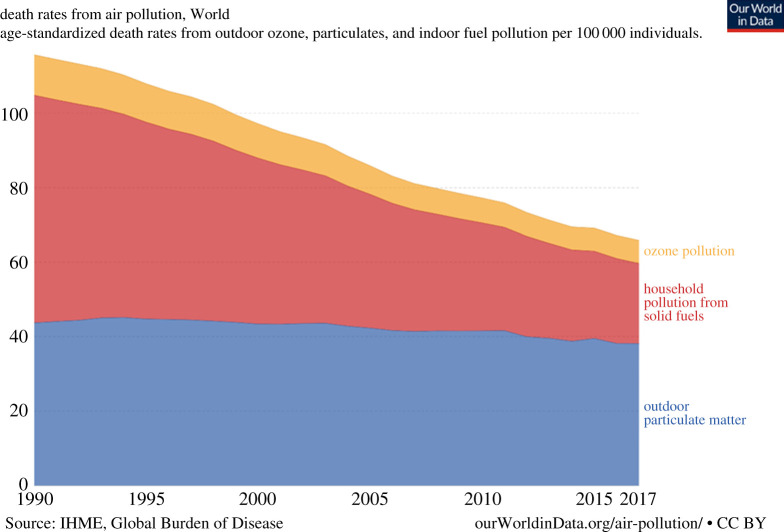
Annual death rates attributed to outdoor PM_2.5_, outdoor ground-level ozone and indoor pollution from solid fuels 1990–2017. Source: www.ourworldindata.org/air-pollution/ based on data from the Global Burden of Disease project. (Online version in colour.)

Despite the widespread elevated levels of PM_2.5_ illustrated in figure 5, data from the global burden of disease project (figure 8) indicate that globally the world may now be on a downward trend of death rates from outdoor PM_2.5_ and from ground-level ozone.

Given the current scale of effects of air pollution on human health and ecosystems and uncertainties in measurements and modelling, it is premature to celebrate the downturn in global emissions of two of the most important air pollutants (SO_2_ and NO*_x_*). However, the temporal pattern in emissions of pollutants displayed in the Environmental Kuznets Curve [120], with increasing efforts to control emissions as economies mature, continues to be consistent with observations as parts of Asia now show substantial reductions in emissions, at least for pollution arising from combustion sources. Furthermore, there is a reasonable expectation that measures to combat climate change and increase the use of renewable energy in developing regions, especially Africa, may substantially mitigate emissions of air pollutants as their economies develop.

## 18. Concluding remarks

In providing a chronology of what has become a very large and complex field, this narrative has, of necessity, been selective. It has also been excessively brief on developments over the last two decades during which the range of issues, geographical scale and very different trends in different areas of the world obscures the wider picture. The suggestion that the world has passed the peak in air pollution problems is a strong statement, and may prove to be incorrect. However, the evidence from SO_2_ and NO*_x_* emissions is persuasive for the major emitting countries in Europe, North America and also for East Asia. If similarly strong controls were applied to ammonia emissions, which are certainly possible technically, the current problems with the nitrogen cycle could also be addressed [121]. It is less clear when or how global VOC emissions may be controlled, but these emissions will become less important if the world moves towards a lower NO*_x_* chemical climate.

## 19. COVID-19

The Discussion meeting at the Royal Society in London 11th and 12th November 2019 took place just before the first case of the Sars-COVID-19 was reported in China on the 17th November 2019. By June 2020, 6.3 million cases had been reported across 188 countries and territories resulting in 376 000 deaths. Lockdown measures have led to major effects on industrial and transport activities and reduced emissions of many of the primary pollutants contributing to poor air quality. While it is too soon to provide a detailed analysis, there are many preliminary reports, including surface measurements from monitoring networks and satellite remote sensing. In the major cities, reduced combustion-related emissions are revealed by CO_2_ flux measurements, with reductions of 55% in central London [122]. The reductions in urban NO_2_ in the UK during the first weeks of lockdown of 20–30% [122] are similar to reductions in other major cities across the developed world. Similar reductions have been observed in column NO_2_ satellite remote sensing (J.P. Burrows, personal communication, 2020).

The effects on PM_2,5_ are much smaller and more variable than effects on NO_2_, with some COVID-19-affected cities in China reporting reductions in PM_10_ similar in scale to reductions in NO_2_ but for a shorter period [123]. The analysis for the UK during lockdown suggested reductions in personal exposure in London to PM_2.5_ in the range 5–25%, depending on the mode of travel, but effects on ambient PM are small and very variable.

The global scale of the pandemic produced a clear effect on global emissions of combustion-related emissions of pollutants, with expected health and environmental benefits due mainly to reduced NO*_x_* emissions. Whether these benefits lead to longer-term reductions in emissions is much less clear as transport and industrial emissions grow following the widespread population lockdown. It seems likely that an effect of COVID-19 will be to reduce net acidity and increase the gaseous alkaline fraction [16] as transport and combustion emission are reduced, but with little anticipated reduction in NH_3_ emissions from agriculture. While this may be associated with health benefits, additional adverse effects of ‘alkaline air’ on ecosystems will also need to be considered.

## Data accessibility

The bulk of the paper is text and review, but there are data provided for this paper which have not been published. The data are shown in figures 2, 4 and 6. The sources for the data are listed in the legends for each figure and the data may be obtained from the named sources.
